# Exercise Intensity and Pacing Pattern During a Cross-Country Olympic Mountain Bike Race

**DOI:** 10.3389/fphys.2021.702415

**Published:** 2021-07-19

**Authors:** Steffan Næss, Ove Sollie, Øyvind Nøstdahl Gløersen, Thomas Losnegard

**Affiliations:** Department of Physical Performance, Norwegian School of Sports Sciences, Oslo, Norway

**Keywords:** mountain bike cycling, critical power, anaerobic capacity, maximal aerobic power, intermittent exercise intensity, pacing strategy

## Abstract

**Objective:** To examine the power profiles and pacing patterns in relation to critical power (CP) and maximal aerobic power (MAP) output during a cross-country Olympic (XCO) mountain bike race.

**Methods:** Five male and two female national competitive XCO cyclists completed a UCI Cat. 1 XCO race. The races were 19 km and 23 km and contained five (female) and six (male) laps, respectively. Power output (PO) during the race was measured with the cyclists’ personal power meters. On two laboratory tests using their own bikes and power meters, CP and work capacity above CP (*W'*) were calculated using three time trials of 12, 7, and 3 min, while MAP was established based on a 3-step submaximal test and the maximal oxygen uptake from the 7-min time trial.

**Results:** Mean PO over the race duration (96 ± 7 min) corresponded to 76 ± 9% of CP and 63 ± 4% of MAP. 40 ± 8% of race time was spent with PO > CP, and the mean duration and magnitude of the bouts >CP was ~8 s and ~120% of CP. From the first to last lap, time >CP and accumulated *W*' per lap decreased with 9 ± 6% and 45 ± 17%, respectively. For single >CP bouts, mean magnitude and mean *W*' expended decreased by 25 ± 8% and 38 ± 15% from the first to the last lap, respectively. Number and duration of bouts did not change significantly between laps.

**Conclusion:** The highly variable pacing pattern in XCO implies the need for rapid changes in metabolic power output, as a result of numerous separate short-lived >CP actions which decrease in magnitude in later laps, but with little lap-to-lap variation in number and duration.

## Introduction

Cross-country Olympic (XCO) mountain biking races are off-road cycling events characterized by high-intensity mass-starts and undulating terrain. The varied track structure leads to a high workload variability, with numerous bursts of high power output (PO; Inoue et al., 2012; Macdermid and Stannard, 2012; Granier et al., 2018; Hays et al., 2018). Such events challenge the contestants’ ability to optimally distribute effort throughout a race, resulting in multiple physiological characteristics being highlighted when attempting to explain what affects performance in the sport (Impellizzeri et al., 2005b; Abbiss and Laursen, 2008; Macdermid and Stannard, 2012; Abbiss et al., 2013; Hays et al., 2018).

Several studies have shown the importance of aerobic characteristics for performance, such as maximal oxygen uptake (V̇O_2max_; Impellizzeri and Marcora, 2007; Hays et al., 2018), maximal aerobic power (MAP) output (Granier et al., 2018; Hays et al., 2018), and ventilatory and lactate thresholds (Impellizzeri et al., 2005a; Miller et al., 2014; Miller and Macdermid, 2015; Viana et al., 2018). Anaerobic power has also been classified as an important determinant of performance because of the intermittent aspect of XCO racing (Inoue et al., 2012), but to date, there have been limited studies conducted.

Although lap-to-lap pacing and physiological performance predictors have been extensively studied (Impellizzeri et al., 2005a,b; Gregory et al., 2007; Abbiss et al., 2013), the intralap variability is not well accounted for Inoue et al. (2012) and Novak et al. (2018). In addition, laboratory tests of aerobic fitness are not readily available to coaches and athletes due to the need for advanced equipment. Noticing the lack of an ecological testing method, Miller and Macdermid (2015) proposed critical power (CP) as an ecological framework to assess performance in XCO. CP is considered to be the highest sustainable rate of oxidative metabolism without a continuous loss of homeostasis, defined as the boundary between steady state and non-steady state exercise (Poole et al., 1988; Jones et al., 2008). Applying the CP concept to XCO could thus delineate work attributable to aerobic and anaerobic energy sources during a race (Chidnok et al., 2012; Martin et al., 2012; Hays et al., 2018).

In intermittent exercise such as XCO, PO > CP is believed to drain a finite capacity, termed *W*', to perform work above CP. *W*' is thought to reflect mainly anaerobic energy stores (Poole et al., 2016). It has been demonstrated that *W*' can be regenerated when PO < CP (Coats et al., 2003; Chidnok et al., 2012); however, the regenerating kinetics are unclear (Bartram et al., 2018; Chorley et al., 2019; Vassallo et al., 2020). Interestingly, recent studies on the pacing patterns of XCO have displayed a shift in the contribution of actions above MAP, suggesting anaerobic actions decrease throughout an event (Granier et al., 2018; Hays et al., 2018). Furthermore, multiple studies have suggested that the mean PO during a race may not adequately describe the metabolic energy demand, in part due to technically challenging downhill segments where PO is zero (Hurst et al., 2012; Miller et al., 2017; Hays et al., 2018). Taken together, although both technical and physical abilities affect XCO performance, *W'* availability could affect high-intensity bouts during a race (Chidnok et al., 2012; Hays et al., 2018). However, this is yet not well investigated in intermittent endurance sports.

Assessing XCO races using the CP framework could elucidate how anaerobic capacity is used throughout a race and provides a practical test for assessing performance that can be used by coaches and athletes in the sport (Chidnok et al., 2012; Triska et al., 2017). To the best of our knowledge, no studies have applied the CP concept to examine the workload requirements of an XCO race. Consequently, the aim of this study was to assess the workload requirements of XCO races using continuous measurements of power output during a race, with special emphasis on > CP actions. We hypothesized that mean power output during the race would be close to each cyclist’s CP and that *W'* expended per bout would decrease as a function of race time.

## Materials and Methods

### Participants

Seven competitive XCO cyclists were recruited for this study (female *n* = 2), all competing at a national standard in Norway [Age: 23.4 years (range: 19–31); mass 68.5 kg (range: 61–88); height 1.77 m (range: 1.61–1.98)]. Inclusion criteria were as follows: (1) participation in a specified national XCO race and (2) access to a portable power meter for use during the race and post-race testing. Before data collection, each cyclist gave written informed consent to participate in the study. The protocol was approved by the local ethics committee of the Norwegian School of Sport Sciences and reported to the Norwegian Centre for Research Data.

### General Design

The study consisted of three sessions. First, cyclists took part in a XCO race before reporting to the laboratory on two occasions separated by at least 48 h, the first of which served as familiarization. The men and women competed in different races, but on the same track, at the same time of day. In the indoor testing sessions, the cyclists completed a submaximal incremental exercise test, a 30-s maximal all-out cycling test, and three maximum effort time trials of 12 (TT12), 7 (TT7), and 3 (TT3) min. The sessions were used to determine each cyclist’s MAP, Peak PO, VO_2max_, CP, and *W*', where data were recorded in the second session. All indoor tests were performed using their own bikes and power meters mounted on a smart trainer. The same test leaders conducted all tests.

### Race Testing

**Figure 1:**
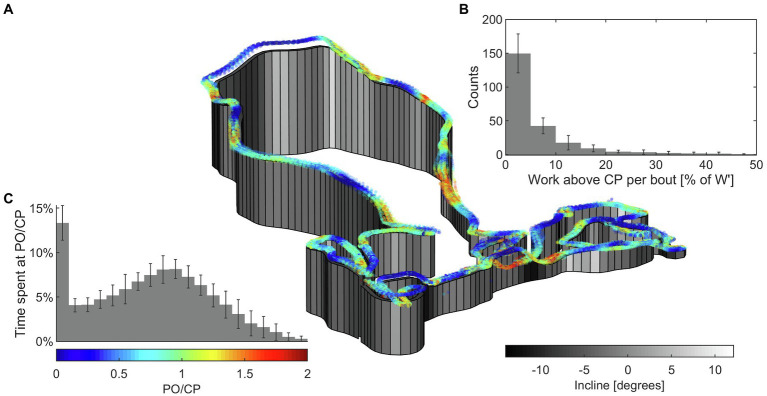
Graphical presentation of power output from the XCO race. **(A)** Topographic illustration of lap path with incline and PO data. Start lap not shown. **(B)** Distribution of bouts above CP sorted by the amount of *W*' expended, normalized to individual *W*'. Count is the number of bouts in each bin throughout the race, per participant. Error bars indicate between-participant SD. **(C)** Distribution of PO normalized to CP throughout the race, displayed as the percentage of race time spent within each bin for an average participant. Error bars indicate between-participant SD.

The cyclists competed in an UCI Cat. 1 XCO race (Karl XII rittet, Norway). A single circuit of the track was 3.8 km and included 101 m climb (Figure 1), with a total race length of 19 km and 23 km for women and men, respectively. Lap times, PO, heart rate (HR), and speed were recorded during the race. The number of laps differed between sexes; six for men and five for women, respectively. To compare data from all cyclists, the races were divided into the following parts: start loop (SL), round 1 (R1), round 2 (R2), round 3(R3), the second to last lap (LN-1), and final lap (LN; Granier et al., 2018). Data were recorded at a frequency of 1 Hz and transmitted to each cyclist’s personal cycling computer.

### Indoor Testing

**Figure 2:**
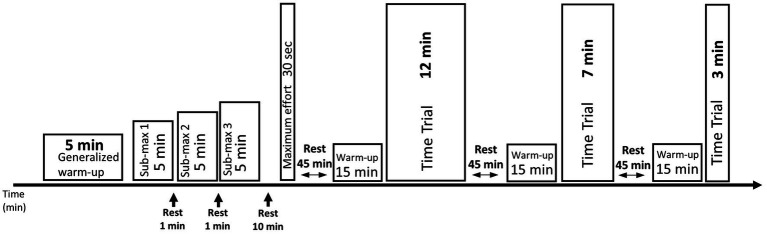
Illustration of test protocol for submaximal workloads and performance tests conducted on the indoor test days on subjects’ personal bikes mounted to the direct-drive smart trainer. Three submaximal workloads were applied; 150, 200, and 250 W for men and 125, 175, and 195 W for women, respectively. This was followed by performance tests beginning with a 30-s all-out test before a 12 min time trial, a 7 min time trial, and a 3 min time trial in that order. All performance tests after the maximal effort test were separated by 45-min rest and 15-min warm-up.

Each cyclist completed two test days, with the first day serving as a familiarization, where individual power-duration relationships were calculated based on PO in the second session (Triska et al., 2017). VO_2_ data for time trials (TT) were only recorded for the second test day. The days were otherwise identical and are shown in Figure 2. Data from all tests were recorded in the second session.

### Submaximal Test

Each cyclist completed a 10-min warm-up (range 50–150 W) before a submaximal, incremental exercise test to determine individual relationships between PO and VO_2_. The test consisted of 5-min steps at work rates of 150, 200, and 250 W for males and 125, 175, and 195 W for females, with 1-min breaks between each of the work rates. The cyclists were free to choose their preferred cadence but were instructed to maintain a constant cadence during all tests. V̇O_2_, respiratory exchange ratio, and HR were measured during the last 2.5 min as well as the cyclist’s rate of perceived exhaustion (RPE) using Borg’s 6 to 20 scale at the end of each step (Borg, 1982). The cyclists were instructed to remain seated and maintain a consistent cadence, which would also be used in the time trials. The submaximal work rates corresponded to 46 ± 7, 57 ± 10, and 68 ± 10% of VO_2peak_ achieved during the TT7.

### Performance Tests

Following 10-min active recovery (range 50–200 W), a 30-s maximal all-out cycling test, starting from a standstill, was performed to determine the maximal power output for each cyclist. A 5-s countdown initiated the test and cyclists were strongly encouraged to accelerate as much as possible and maintain maximal power output. Subjects were instructed to remain seated throughout the test and were free to change the ergometer resistance on the tablet throughout the test. After the tests, they were allowed up to 10-min active recovery before the 45-min rest period prior to the remaining performance test.

CP and *W'* were established using TT12, TT7, and TT3 in that order (Karsten et al., 2017). Before each TT, a 15-min warm-up was completed with light intensity (<200 W for males and <150 W for females) from start to 7 min, followed by medium-high intensity from min 7 to 10, with participants instructed to end at 15 RPE, and finishing with another 5-min’ light intensity from min 10 to 15. Time trials started from a standstill and the cyclists were free to adjust the ergometer resistance throughout the test except for the first min of TT12 and TT7, and for 30 s for TT3, to avoid overly aggressive pacing strategies. Specifically, the initial PO was set to the mean PO from the corresponding test on the familiarization day. The cyclists were instructed to maintain their previously chosen cadence throughout the TT. V̇O_2_ was recorded throughout the tests. Feedback on time remaining, and strong verbal encouragement, was provided during the test. After each TT, the cyclists were allowed 10 min of active light intensity recovery before proceeding to 45 min of passive rest.

### Apparatuses

During the race and indoor testing, participants used their personal bike, HR monitor, cycling computer, and power meter. Two different power meters were in use; Quark (*N* = 6; Quarq, Spearfish, SD, United States) and 4iii (*N* = 1; Cochrane, Alberta, Canada). Oxygen consumption was measured with an automatic ergospirometry system, with a mixing chamber set up, on the second day (Oxycon Pro, Jaeger Instrument, Hoechberg, Germany). Heart rate was recorded with each participant’s personal monitor.

All participants used their own bike and power meter mounted on a direct-drive smart trainer (KICKR, Wahoo Fitness, Atlanta, United States) and controlled using the accompanied app (Wahoo Fitness, 2019, version 5.23.0). The trainer’s reliability has been assessed previously (Miller et al., 2016). A tablet was mounted close to the handlebars, where the participants were able to adjust their work rate on self-paced tests. Participants were instructed to use the power and cadence feedback from their personal cycling computer to gauge their efforts during all tests.

### Data Analysis and Statistics

Linear least squares regression was used to identify CP and *W'* using the model

$$P=W'\left[\frac{1}{t}\right]+CP,$$

where [1/*t*] is the inverse of time. VO_2_ and PO were used to assess work during submaximal workloads from which linear regression (without a forced y-intercept) was used to extrapolate the power corresponding to MAP. Power meter data were imported to Golden Cheetah (Golden Cheetah training software, goldencheetah.org) and subsequently exported to Microsoft Office Excel 365 (Microsoft, Redmond, United States) and MATLAB R2019a (MathWorks, Natick, Massachusetts, United States) for further analysis.

Data included in statistical analyses were checked for normality with a Shapiro-Wilk test. Unless otherwise specified, data are displayed as mean and standard deviation (SD), lap-to-lap changes in PO are displayed as mean with 95% confidence intervals (95% CI). Male and female athletes were initially investigated independently but no difference in parameters related to CP bouts were found, and both displayed similar trends throughout the race. Due to the small sample size and similarities, males and females were analyzed as a combined group. One-way repeated ANOVAs with Bonferroni correction for multiple comparisons were performed to identify statistically significant differences between laps using accumulated *W'* per lap, mean *W'* expended per bout > CP, mean duration per bout > CP, mean magnitude per bout > CP, percent of race time spent above MAP, speed, mean PO, CAD, and mean HR as dependent variables. Effect sizes (ES) were calculated using Hedge’s *g* (small *g* = 0.2; moderate *g* = 0.5; large *g* = 0.8; and very large *g* = 1.3; Sullivan and Feinn, 2012; Ferguson, 2016). Sphericity was checked using Mauchly’s test of sphericity, and if the assumption of sphericity had been violated the Greenhouse-Geisser correction was used. The statistical significance level was set at *p* < 0.05, and data were analyzed and graphically presented using Prism 9 (GraphPad Software, San Diego, CA, United States).

## Results

Race characteristics including duration, lap time, mean HR, mean CAD, and mean speed, as well as peak, relative, and mean PO are shown in Table 1.

**Table 1:** Characteristics from the race and subject characteristics from indoor testing (*N* = 7, of which men *N* = 5 and women *N* = 2).

	Measure	Mean ± SD	Range
Subject characteristics	VO_2max_ (ml × min^−1^ × kg^−1^)	71 ± 8	59–79
	MAP (W)	397 ± 118	267–568
	CP (W)	329 ± 80	241–456
	*W'* (kJ)	13.7 ± 5.3	8–24.3
Race characteristics	Race duration (min)	96 ± 7	87–107
	Avg. lap time (min)	16 ± 2	14–20
	Avg. Power output (W)	249 ± 63	153–320
	Relative power output (W·kg^−1^)	3.6 ± 0.7	2.5–4.4
	Avg. speed (km·h^−1^)	14.4 ± 1.9	9.7–18.2
	Peak power (W)	1,087 ± 266	692–1,404
	Avg. HR (bpm)	180 ± 4	170–191
	Avg. CAD (rpm)	66 ± 3	59–72

*Presented with Mean ± SD and range. MAP, maximal aerobic power; CP, critical power; km·h^−1^, kilometers per hour; HR, heart rate; and CAD, cadence*.

**Figure 3:**
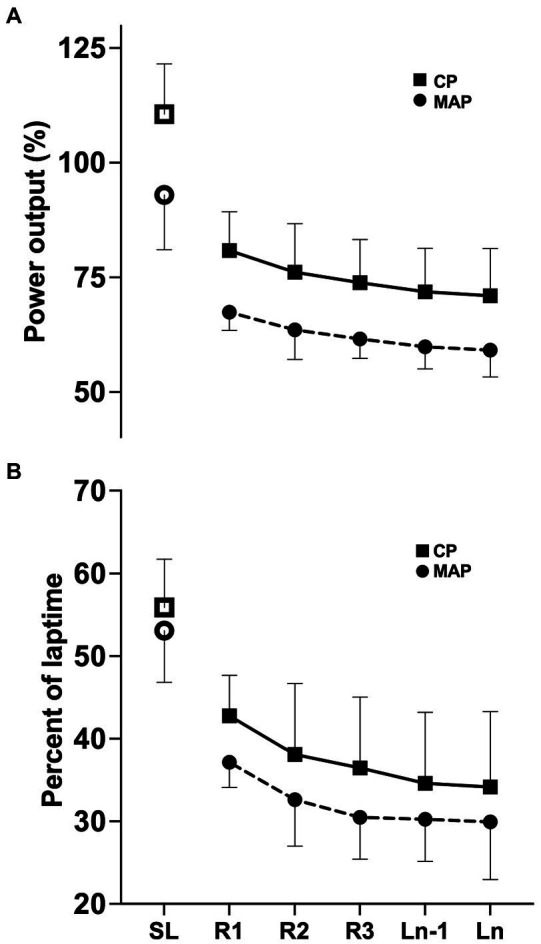
Lap by lap measurements for: **(A)** power output in percent of individual CP (squares) and MAP (circles) and **(B)** percent of lap time above CP (squares) and MAP (circles). SL is displayed as an open icon, separate from following rounds. Data are presented as mean ± 95% CI.

Lap-to-lap mean PO normalized to individual CP and MAP is presented in Figure 3A with 95% confidence intervals (95% CI). A graphical presentation of power output during part of the race is presented in Figure 1, displaying considerable fluctuations in power output. There was a difference in mean PO between laps [*F* (2.143, 12.86) = 10.85, *p* = 0.002], decreasing from R1 to LN-1 (*p* = 0.022; *g* = 0.43) and LN (*p* = 0.016; *g* = 0.45) and from R2 to LN (*p* = 0.012; *g =* 0.24). Speed followed a similar pattern to mean PO [*F* (2.139, 10.69) = 17.34, *p* < 0.001].

Mean PO over the race duration corresponded to 76 ± 9% of CP and 63 ± 4% of MAP. Time spent with zero PO (i.e., not pedaling) was 20.5 ± 3.1 min or 27 ± 3% of total race time. PO was highly variable during the race, ranging from 0 to 331 ± 43% of CP and 0 to 277 ± 29% of MAP.

**Figure 4:**
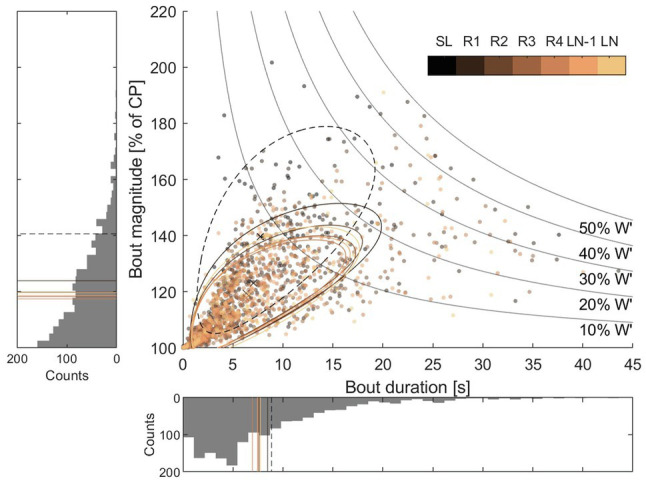
Scatterplot and histograms showing the distribution of duration and magnitude of > CP bouts. Data from all participants have been aggregated. Different colors denote different laps, as shown in the color bar in the upper right corner. The histograms show data aggregated for all laps; the seven lines indicate mean values for different laps. Dotted lines indicate the start lap (SL), which was different from succeeding laps. There was a decline in bout magnitude as lap number increased; however, there was no significant decrease in bout duration (see Table 2 for details). The twisted ellipsoid regions in the scatterplot show the approximate distribution of bout duration and magnitude for the different laps. They were obtained by fitting an ellipse to the square-root transformed distributions of bout durations and magnitudes. Each twisted ellipsoid contains approximately 68% of the bouts for a given lap.

The percentages of race time spent > CP and > MAP were 40 ± 8% and 26 ± 8%, respectively. The percentages of lap time spent > CP and > MAP are displayed in Figure 3B, showing changes between laps in both CP [*F*(1.874, 11.25) = 8.053, *p* = 0.007] and MAP [*F*(1.955, 11.73) = 7.768, *p* = 0.007]. Figure 4 displays bout duration and magnitude along with percentage of *W'* expended in each bout.

Lap by lap comparison of accumulated *W*' expended, mean *W*' expended per bout, mean > CP bout duration, and magnitude are presented in Table 2. Accumulated *W*' expended changed between laps [*F*(2.009, 12.05) = 20.25, *p* < 0.001], with a decrease from R1 to the last three laps (R3, *p* = 0.007, *g* = 1.22; LN-1, *p* = 0.018, *g* = 1.46; and LN, *p* = 0.014, *g* = 1.48). This is consistent with the mean *W*' expended per > CP bout, which had a similar decrease [*F*(2.157, 12.94) = 15.57, *p* < 0.001]. However, while the mean > CP bout magnitude decreased from R1 to all later laps [*F*(2.411, 14.46) = 18.25, *p* < 0.001], the mean > CP bout duration did not change between laps [*F*(2.004, 12.02) = 3.524, *p* < 0.062].

**Table 2:** Lap by lap comparisons of factors related to bouts > CP.

Round	Accumulated *W'* per lap (Fraction of *W'*)		Mean *W'* expended per bout (Fraction of *W'*)		Mean bout duration (s)		Mean bout magnitude (Fraction of CP)		Number of bouts above CP	
	Mean ± SD	Range (min–max)	Mean ± SD	Range (min–max)	Mean ± SD	Range (min–max)	Mean ± SD	Range (min–max)	Mean ± SD	Range (min–max)
SL	1.07 ± 0.31	0.59–1.43	0.113 ± 0.032	0.059–0.143	8.9 ± 1.5	6.7–11.5	1.41 ± 0.07	1.32–1.5	17 ± 3	14–22
R1	3.08 ± 0.93	1.74–4.37	0.072 ± 0.021	0.045–0.104	8.5 ± 1.2	7–10.6	1.24 ± 0.05	1.17–1.33	92 ± 12	73–112
R2	2.17 ± 0.97	1.08–3.77	0.054 ± 0.022	0.030–0.094	7.6 ± 1.5	5.8–10.5	1.19 ± 0.06^†^^,^^##^	1.12–1.32	89 ± 11	75–111
R3	1.95 ± 0.94^†^^,^^###^	0.88–3.3	0.048 ± 0.022^†^^,^^‡^^,^^#^^,^^##^^,^^*^	0.024–0.089	7.5 ± 1.9	5.4–11.6	1.18 ± 0.06^†^^,^^###^	1.11–1.3	90 ± 11	69–108
Ln-1	1.83 ± 0.78^†^^,^^####^	0.99–3.17	0.049 ± 0.018	0.025–0.083	7.6 ± 1.6	5.2–10.7	1.19 ± 0.05^†^^,^^###^	1.11–1.29	88 ± 10	75–102
Ln	1.74 ± 0.89^†^^,^^####^	0.77–3.37	0.045 ± 0.020^†^^,^^####^	0.022–0.082	7.2 ± 1.4	5.2–9.8	1.18 ± 0.06^†^^,^^###^	1.11–1.29	88 ± 9	76–104

*Data are presented with Mean ± SD and range. SL, start loop; R1, round 1; R2, round 2; R3, round 3; Ln-1, penultimate lap; and Ln, last lap*.

† *Significantly different from R1.*

‡ *Significantly different from R2.*

# *Effect size for difference with the first lap.*

* Effect size for difference with the second lap (^#^ or ^*^, small;

## , *moderate;*

### , *large; and*

#### , *very large)*.

## Discussion

The present study examined the power profiles and pacing patterns in XCO in relation to critical power (CP) and MAP. The main findings from this study are as follows:

- About 40% of race time was spent above the CP and 26% above MAP, with bouts on average lasting for 8 s and exceeding CP by 20%.
- Work above CP during each individual bout was small compared to *W'* and decreased from on average 11% of *W'* during the start lap to 3–5% during the final laps. This was explained by a decreased bout magnitude from early to later laps, while bout duration and number did not change between laps.

To the best of our knowledge, this is the first study to use the CP concept to understand the work requirements of XCO. These findings corroborate previous studies showing a fast start strategy followed by a more even pacing pattern on a lap-to-lap basis (Abbiss et al., 2013; Granier et al., 2018; Viana et al., 2018).

The decrease in percentage of lap time spent > CP was accompanied by a lower accumulated *W′* expended, with mean bout magnitude being significantly higher in R1 than subsequent laps, and small or trivial changes in mean bout duration and number of bouts (Table 2). Progressive fatigue development could explain the gradual shift toward less intensive PO relative to CP, resisting accumulation of metabolites that could be detrimental to performance (Chidnok et al., 2013; Davies et al., 2017; Hays et al., 2018). Indeed, earlier studies have demonstrated that an intermittent workload allows for a greater amount of work done > CP than does a constant workload (Skiba et al., 2012; Chidnok et al., 2013; Chorley et al., 2020), as long as workloads < CP are available for recovery of *W*'. The observations from this study showed frequent bouts with various degrees of *W'* expenditure (Figures 1B, 4), tending to a lower degree of *W'* expended in later rounds (Figure 4; Table 2). The mechanisms, and even the descriptive kinetics, of *W'* recovery are still to be clarified, but possibly reflect a complex interplay of biochemical pathways, including restoration of blood and muscle oxygen stores, high-energy phosphates, and the ability to proceed with anaerobic glycolysis (Miura et al., 2000; Jones et al., 2010; Chidnok et al., 2013; Chorley and Lamb, 2020). Recent evidence points toward a bi-phasic recovery, enabling rapid recovery of one fraction of *W*', followed by a slower recovery of the remaining fraction (Caen et al., 2021). Moreover, a progressive decrease in *W'* recovery effectiveness during intermittent exercise has recently been suggested, which could account for the decrease in degree of *W'* expenditure between rounds (Figure 4; Chorley et al., 2019, 2020). As discussed by Gløersen et al. (2020), these two points may explain why athletes in intermittent endurance sports typically choose to expend only a small fraction of their anaerobic energy resources within a supra-maximal bout. Interestingly, any notable changes in number of bouts and the mean duration of bouts did not follow the decrease in accumulated *W'* per lap and mean bout magnitude (Table 2). The lack of a meaningful change in bout duration and number suggests high PO actions are a result of challenges imposed by the track, in support of Martin et al. (2012) conclusions about a spontaneous relationship between pacing and terrain, with even lap-to-lap pacing despite high intralap variability.

Another aspect could be that the decrease in mean bout magnitude is simply a response to the reduced density of riders after the mass-start (Granier et al., 2018). While the mean *W'* expended per bout and accumulated *W*' expended tended to decrease, mean bout magnitude and duration appeared to stabilize after the initial laps (Table 2). XCO cyclists employ an aggressive start to best position themselves relative to other competitors and further maintain an early advantage to allow for easier optimization of performance without the hindrance of other cyclists (Granier et al., 2018). Moreover, the role of decision making for performance suggests that removing the stressor of other competitors could potentially allow for more optimized descents (Miller et al., 2017; Novak et al., 2018), limiting work lost to braking, and reacceleration (Miller et al., 2018, 2019). However, our results show that mean PO declined from initial to later laps; hence, even with a more even intralap pacing, the cyclists were unable to maintain a constant mean lap PO (Figure 3). This indicates that elements besides rider-density are at work; likely a fatigue component, as similar tendencies have been reported in earlier studies (Granier et al., 2018; Hays et al., 2018; Viana et al., 2018). Exercise assessment models, such as CP, have been proposed to drift during prolonged exercise and thereby lead to inaccuracies when applied to prolonged durations (Maunder et al., 2021). While little to no change in calculated CP after 80-min heavy intensity exercise has been reported (Clark et al., 2019a), continuous exercise approaching 2 h has been shown to be detrimental to both CP and *W*', implying a fatigue component unaccounted for by the current CP calculations (Clark et al., 2018, 2019a,b). A drift in CP could influence the observed pacing and present a “durability” parameter, understood as extending the time to a detrimental shift in CP, but it is uncertain how this affects the intermittent nature of XCO. Other factors are also likely to be involved in the overall intensity, i.e., mental fatigue (Salam et al., 2018) or the characteristics of high-intensity bouts (de Souza et al., 2016; Schäfer et al., 2019). Ultimately, the aggressive initial laps coupled with frequent high-intensity segments presented by the track led to the positive pacing observed in this study, hindering sufficient recovery between high-intensity bouts. However, the precise fatigue, recovery and durability mechanics that drive the decline are still not adequately described (Clark et al., 2019a,b; Maunder et al., 2021).

### Methodological Considerations

This study examined a race that was already a part of the cyclists’ competitive schedule, and we therefore assume they completed the race as planned, appropriately motivated. This could increase the validity of the study. There was a limited number of potential participants in the study, resulting in heterogeneity in the recruited group, which may have resulted in a broader range of values for PO, speed, and CP parameters (Impellizzeri et al., 2005b). However, it is unlikely to affect our findings because relative individual values were used.

To increase generalizability to PO from the field, the cyclists performed indoor tests on the bike and power meter they used during the race, attached to a smart trainer. While this may limit the potential direct comparison between athletes, we believe it could eliminate some of the error associated with using multiple power meters for field data compared to a standardized indoor ergometer. However, indoor tests in laboratory conditions could result in a lower PO during CP tests and therefore underestimate CP, resulting in an overestimation of CP parameters during the race (Mieras et al., 2014). Furthermore, the methods for calculating CP, validated for field-testing and accompanied by sufficient familiarization trials, are practical to complete with a timer and power meter and may therefore be of more use for coaches and athletes (Karsten et al., 2015, 2017; Triska et al., 2017). While the method has been reported to be reliable and valid, the effects of randomizing the order of time trials have not been evaluated and should therefore not be excluded as a potential factor in the calculation. The method has displayed large variations in *W'* and should therefore be interpreted cautiously.

Difference between sexes in the XCO format has not been elucidated and can therefore not be excluded as a potential limitation, although similar relative characteristics have been suggested (do Carmo et al., 2020; Prinz et al., 2021). However, differences in fatigability between sexes during intermittent exercise and > CP intensity have been reported, and it is not clear how this relates to expression of relative CP parameters during XCO (Billaut and Bishop, 2009; Ansdell et al., 2019, 2020). Conversely, the observed similar progression of relative CP parameters regardless of sex in this study is in line with Prinz et al. (2021), who reported similar behavior of above MAP segments between sexes during XCO races. Ultimately, current research regarding sex differences remains inconclusive and should be considered with caution when applying the CP model to XCO.

### Practical Applications

Recording PO data in XCO are common, although practical tools to analyze performance are limited. Consequently, the ability to use CP could provide a more practical method to analyze the fluctuating workload during racing, with this study presenting bout duration, magnitude, and *W*' expended as potential parameters to describe the demands of XCO racing (Triska et al., 2017, 2018; Hays et al., 2018; Klitzke Borszcz et al., 2019). The results from this study suggest training components targeting frequent bouts > CP could be beneficial, optimizing XCO training with PO variation within intervals (Bossi et al., 2020), sprints, and short intervals (Rønnestad et al., 2015; Inoue et al., 2016; Hays et al., 2021). However, future studies should illuminate the effects of the CP and *W*' parameters on performance to increase applicability for coaches and athletes.

## Conclusion

Cross-country Olympic races elicit frequent work bouts above CP with a highly variable pacing pattern within laps. About 40 ± 8% of race time was spent above CP and 26 ± 8% above MAP, characterized by bouts on average lasting for 8 s and exceeding CP by 20%. Work above CP during each bout decreased from 11 ± 3% of *W'* during the start lap to 3–5% during the final laps. This was explained by a decreased bout magnitude from early to later laps, with trivial changes in number and duration, resulting in a positive pacing pattern. The ability to resist a decrease in bout magnitude balancing the > CP and < CP segments seems an important ability for XCO cyclists.

## Data Availability Statement

The raw data supporting the conclusions of this article will be made available by the authors, without undue reservation.

## Ethics Statement

The studies involving human participants were reviewed and approved by the Ethical Committee, Norwegian School of Sport sciences. The patients/participants provided their written informed consent to participate in this study.

## Author Contributions

SN, OS, ØG, and TL analyzed the data. All authors contributed to design of the study, collection of the data, manuscript revision, and approved the submitted version.

### Conflict of Interest

The authors declare that the research was conducted in the absence of any commercial or financial relationships that could be construed as a potential conflict of interest.
